# Thermal Springs

**Published:** 1858-03

**Authors:** 


THERMAL SPRINGS.—An inquiry into the causes of the increased temperature of subterranean waters, and of the springs which they supply, is one of great geological interest, and forms, at the same time, an important portion of the natural history of mineral springs. The question of thermalization may indeed be studied separately from that of mineralization; but, for the most part, the two are blended, the one with the other.

Shall we, with Fourier, admit three constant causes of the temperature of the globe which we inhabit? These are—1. The sun's rays, the unequal distribution of which produces the diversity of seasons and climates. 2. The radiation of caloric and light from innumerable celestial bodies which surround the solar system. This is the temperature of the planets. 3. The primitive heat which the earth contained when the planets were first formed, and a part of which it retains in the interior of its mass. We shall concern ourselves on this occasion with this last source of terrestrial heat. The form of the spheroid which we inhabit, the regular distribution of the inferior strata, made manifest by the experiments with the pendulum, their density increasing with their depth, and various other considerations tend to prove Fourier's opinion,—viz., that a very intense heat has pervaded all parts of the globe. This heat was dissipated by radiation into surrounding space, the temperature of which, beyond the common atmosphere, is much lower than that of the freezing point of water. The mathematical expression of the law of refrigeration, as announced by Fourier, shows that the primitive heat contained in a spheroid mass of such large dimensions as the earth, diminishes more rapidly at the surface than at the parts situated at a great depth. These last preserve nearly all their heat for a wonderfully long period.

Experimental observations give full support to this theory, by their showing that the heat, after a certain distance beneath the surface or below the line of invariable temperature, dependent on the sun's rays, increases regularly with increase of depth. Thermometrical observations show, also, that the actual distribution of heat in the crust of the earth is precisely that which would take place if the globe had been formed in a medium of a very high temperature, and then subsequently cooled.

It may be made a question, how long and how far may the cooling process be expected to go on in the interior; and to what extent does the caloric escape from the central focus, and is radiated from the earth's surface? Resting his deduction on observations made of the time taken for spheroidal bodies of a high temperature to cool, Fourier asserts that thirty thousand years must elapse before the present rate of increase, which he estimates to be equal to one-thirtieth of a degree of the centigrade thermometer, or a little more than a sixteenth of a degree of Fahrenheit, for every metre, or three feet, three inches, four lines in depth, would be reduced to one-half of what it is at present. From the time of the Greek school of Alexandria to the present, a period of upwards of two thousand years, the temperature of the surface of the earth has not been diminished, by radiation of the heat reaching it from the interior, by more than the 300th part of a degree of Fahrenheit.

There is quite a uniformity in the general results of observations made in the mines of Brittany, Saxony, England, Mexico, and Peru, whether they are of silver, copper, tin, coal, or salt; all going to prove an increase of temperature with that of the depth. This is manifested both in the galleries of the mines and in the springs rising in them, and also in the rocks composing their sides. These great diversities of geological and minerological characters indicate clearly a general cause, quite independent of any local accumulations, such as the presence of pyrites, coal formations, etc. Pointing clearly to the same conclusions are the thermometrical records in the case of artesian wells; and, above all, the volcanic activity of the earth in ejecting molten masses from opened clefts or fissures.

From these premises respecting the causes of the temperature of the earth we readily deduce the inference that the cold or common springs, and those of a more elevated temperature, or thermal ones, constitute two systems; the former deriving their temperature from that portion of the earth which is under solar influence; the latter obtaining theirs from the deeper seated and probably central heat. Cold springs, from this showing, will be found to be in relation with the upper strata, and the thermal ones of a high heat, with the inferior or deeper strata.

The common springs of a country, although undergoing some fluctuations of temperature coincident with those of the different seasons, approach very nearly to the annual mean of the temperature of the soil, and also of the atmosphere. Hence the temperature of the common springs is often taken, not only as an exponent, but as an equivalent of that of any given spot, of which we have not had opportunity for keeping a thermometrical record, for a sufficiently long period, to allow of our ascertaining, in this way, the mean for the year. This mean is also represented by the line of invariable temperature at a certain depth beneath the surface, which may be considered as the limit of the passage of solar heat from the surface, and of that of internal heat from below. This limit must naturally vary in its depth, according to the degree and duration of the atmospheric heat, the nature of the soil and of the strata, and the elevation above the ocean, of the spot where the observation is made. At the equator, this line will be only a foot or so below the surface, while at Paris, in N. lat. 48° 50′, it is very little under 93 feet. If we imagine a curve drawn from the equator to the poles, all the intermediate points of which should have the same temperature, and take the mean of that on the equator at 81° F., as a starting point, it will be very little beneath the surface there; but it will be deflected downward more and more as the latitude increases; and in the Arctic circle or near the poles it will be at the greatest depth in the earth. This curve, if it were to pass through Northumberland, (Eng.,) would there dip as low as 1365 feet; for at this depth the waters below the bottom of the mines have the mean temperature of the equator. Near Paris, again, the curve would go much deeper to reach the same temperature, if we were to form an opinion from that of the artesian well of Grenelle, which, at a depth of 1800 feet below the surface, has a temperature of 82·4° F.

To the class of imaginary subterranean lines of this kind the term *Isogeothermal* has been applied by Kupffer, and *Chthonisthermal*, or *Subterranean Isothermal Lines*, by Bischoff. A series of these, while they would indicate the increasing temperature of the earth with the augmentation of distance from the surface, could not be expected to be parallel to the surface or to each other. They must undergo deflections similar to those of the isothermal lines which indicate the mean temperature of the surface and incumbent atmosphere of the place which they traverse.

In using the term *thermal* in connection with the water of springs, we give it a much larger significance than is commonly conveyed, since it will include not only hot, and warm, and tepid springs, but also all those which have an average temperature exceeding that of the soil, or of the ordinary cold springs at the level of the spot whence they are emitted. It matters not, therefore, according to this definition, whether the excess be 5° or 50° F., the term thermal is still applicable.

Were we writing an elaborate essay in place of merely offering the more prominent points of the subject, we would inquire into the cause or causes of the internal heat of the earth, of which thermal springs, volcanos, and earthquakes are evidences on a large scale. It will be sufficient, just now, to mention the three theories which divide the schools of geology respecting the origin of the great classes of cosmical phenomena referred to — viz. the *electrical*, the *chemical*, and the *mechanical*. The last theory is most consonant with the larger number of geological and metereological phenomena, and even mathematical calculation; and it enlists in its favor probably the greater number of natural philosophers and geologists, at the head of whom we may name the celebrated La Place. This theory supposes a permanently internal heat of the earth, kept up by a fusion of its central portion on which the crust of the earth rests.

Admitting the progressive increase of temperature as we recede from the surface of the earth, the next question which arises is the rate of this increase. Cordier estimates the average rate, as deduced from observations made in mines and in other ways, to be forty-five feet for every degree of Fahrenheat. We find the average, from observations made on artesian wells in Great Britain, France, and Germany, to be a little over forty-one feet for every degree of Fahrenheat. If we grant Cordier's estimate, and assume the increasing temperature to be continued downward, we should reach the ordinary boiling point of water at about ten miles below the surface; and at the depth of twenty-four miles we should arrive at the melting point of iron, a heat sufficient to fuse almost every known substance, and which, according to the experiments of Daniell, is equal to 2786° Fahr.

The geological affinities of thermal springs will form, on a future occasion, a brief introduction to an account of their geographical distribution. Our collection, made with some care and after a good deal of search, is, we believe, much larger than can be found in any work which treats professedly of mineral and thermal springs.

